# Insulin Rescued MCP-1-Suppressed Cholesterol Efflux to Large HDL2 Particles via ABCA1, ABCG1, SR-BI and PI3K/Akt Activation in Adipocytes

**DOI:** 10.1007/s10557-021-07166-2

**Published:** 2021-03-19

**Authors:** Runlu Sun, Pu Fang, Jieyu Jiang, Canxia Huang, Junjie Wang, Qi Guo, Hongwei Li, Xiaoying Wu, Xiangkun Xie, Yuan Jiang, Qian Chen, Jinlan Bao, Jingfeng Wang, Hong Wang, Yuling Zhang

**Affiliations:** 1 Department of Cardiology, Sun Yat-sen Memorial Hospital, Sun Yat-sen University, No. 107, the West of Yanjiang Road, Yuexiu District, Guangzhou, 510120 China; 2 Guangdong Province Key Laboratory of Arrhythmia and Electrophysiology, Guangzhou, China; 3 Centers for Metabolic & Cardiovascular Research, Department of Pharmacology, Temple University, Medical Education & Research Building, Rm. 1060 3500 North Broad Street, Philadelphia, PA USA; 4 Graceland Medical Center, the Sixth Affiliated Hospital, Sun Yat-sen University, Guangzhou, China; 5 Intensive Care Unit, Sun Yat-sen Memorial Hospital, Sun Yat-sen University, Guangzhou, China; 6 Comprehensive Department, Sun Yat-sen Memorial Hospital, Sun Yat-sen University, Guangzhou, China

**Keywords:** Insulin, MCP-1, Adipocytes, Cholesterol efflux; large HDL2 particles

## Abstract

**Purpose:**

Intracellular cholesterol imbalance plays an important role in adipocyte dysfunction of obesity. However, it is unclear whether obesity induced monocyte chemoattractant protein-1 (MCP-1) causes the adipocyte cholesterol imbalance. In this study, we hypothesize that MCP-1 impairs cholesterol efflux of adipocytes to HDL2 and insulin rescues this process.

**Methods:**

We recruited coronary artery disease (CAD) patients with obesity and overweight to analyze the association between MCP-1 and HDL2-C by Pearson correlation coefficients. We performed [^3^H]-cholesterol efflux assay to demonstrate the effect of MCP-1 and insulin on cholesterol efflux from 3T3-L1 adipocytes to large HDL2 particles. Western blot, RT-qPCR, cell-surface protein assay, and confocal microscopy were performed to determine the regulatory mechanism.

**Results:**

Plasma MCP-1 concentrations were negatively correlated with HDL2-C in CAD patients with obesity and overweight (*r* = −0.60, *p* < 0.001). In differentiated 3T3-L1 adipocytes, MCP-1 reduced cholesterol efflux to large HDL2 particles by 55.4% via decreasing ATP-binding cassette A1 (ABCA1), ABCG1, and scavenger receptor class B type I (SR-BI) expression. Intriguingly, insulin rescued MCP-1 mediated-inhibition of cholesterol efflux to HDL2 in an Akt phosphorylation-dependent manner. The rescue efficacy of insulin was 138.2% for HDL2. Moreover, insulin increased mRNA and protein expression of ABCA1, ABCG1, and SR-BI at both transcriptional and translational levels via the PI3K/Akt activation.

**Conclusions:**

These findings indicate that MCP-1 impairs cholesterol efflux to large HDL2 particles in adipocytes, which is reversed by insulin via the upregulation of ABCA1, ABCG1, and SR-BI. Therefore, insulin might improve cholesterol imbalance by an anti-inflammatory effect in adipocytes. Clinical trial registration number: ChiCTR2000033297; Date of registration: 2020/05/ 27; Retrospectively registered.

**Supplementary Information:**

The online version contains supplementary material available at 10.1007/s10557-021-07166-2.

## Introduction

Obesity is an important feature of metabolic syndrome and is characterized by insulin resistance, glucose intolerance, dyslipidemia, and hypertension [1]. Especially, in CAD subjects, obesity is directly associated with increased mortality [2]. Studies indicate that obesity is associated with excessive infiltrating macrophages and larger adipocytes in adipose tissues, and these factors result in chronic, low-grade adipose inflammation [3, 4].

MCP-1, a chronic inflammatory factor, plays an important role in adipose tissues of obesity [5, 6]. The abundance of MCP-1 in adipose tissues is increased in genetically obese diabetic mice, in WT mice with obesity induced by a high-fat diet [6], and in obese humans [7, 8]. Furthermore, increased MCP-1 expression in adipose tissue under the control of the adipocyte P2 gene promoter contributes to insulin resistance and macrophage accumulation in adipose tissue [5].

Adipose tissue dysfunction is a characteristic of obesity and is an important risk factor for the development of metabolic syndrome [9–11], and the intracellular cholesterol imbalance in adipocytes has been suggested to play an important role in both adipose tissue dysfunction and obesity [9, 12]. Although adipose tissue contains large quantities of cholesterol, the activity of the cholesterol synthetic and catabolic pathway is limited. Therefore, the bidirectional exchange of cholesterol between adipocytes and serum lipoproteins is highly important for cholesterol homeostasis in adipocytes. A large portion of the cholesterol in the adipocytes is acquired from HDL through both SR-BI-dependent and SR-BI-independent mechanisms [9, 13]. However, cholesterol efflux from adipocytes is crucial for cellular cholesterol imbalance. Studies indicate adipocytes express several cholesterol transporters, such as ABCA1 and ABCG1, which mediate cholesterol efflux to apoA-I and HDL [14, 15], respectively, and SR-BI is a bidirectional transporter that also mediates cholesterol efflux to lipoproteins [16].

Studies show that elevated MCP-1 can alter adipocyte function by decreasing insulin-stimulated glucose uptake [7]. Our previous study indicated that MCP-1 inhibits cholesterol efflux to HDL from human coronary artery endothelial cells [17]. However, whether MCP-1 can suppress cholesterol efflux from adipocytes and cause intracellular cholesterol accumulation is unknown. Insulin has been reported to influence cholesterol removal from different cells, but the results have been controversial [18, 19]. In this study, we investigate the impact of insulin, together with ABCA1, ABCG1, and SR-B1, on MCP-1 mediated cholesterol efflux to large HDL2 particles in adipocytes, as well as the regulatory mechanism.

## Materials and Methods

### Regents

Antibodies against ABCA1(Cat#NB100–2068, RRID: AB_535487), ABCG1(Cat# NB400-132SS, RRID: AB_5920491), SR-B1 (Cat# NB 100–908, RRID: AB_526909), and GAPDH (Cat# NB 100–73,063, RRID: AB_ 1,108,728) were purchased from Novus Biologicals Inc. (Littleton, CO). Fluorescent secondary antibodies including Alexa Fluor® 546 goat anti-mouse IgG (Cat# A-11003, RRID: AB_141370) for ABCA1, Alexa Fluor® 488 donkey anti-rabbit (Cat# A-21206, RRID: AB_1535792) for ABCG1, and Alexa Fluor® 633 donkey anti-goat IgG (Cat# A-21082, RRID: AB_141493) for SR-BI, were purchased from Invitrogen (Carlsbad, CA). Antibodies against phospho-Akt and Akt (Cat# 8200, RRID: AB_ 1,658,157) were purchased from Cell Signaling Technology Inc. (Beverly, MA). Wortmannin were purchased from Cell Signaling Technology Inc. Human ApoA-I, Insulin were purchased from Sigma-Aldrich (St. Louis, MO). Human HDL2 was purchased from Cell Biolabs, Inc. (San Diego, CA). HDL2 subfractions were isolated by sequential density gradient ultracentrifugation from healthy human plasma. The traditional ultracentrifugation method has proved the reliable and the simple available procedure for HDL2 isolation. MCP-1 were purchased from R&D Systems (Minneapolis, MN).

### Human CAD Subjects with Overweight and Obesity and Biochemical Tests

The human study protocol was approved by the Medical Ethical Committee of Sun Yat-sen Memorial Hospital at Sun Yat-sen University [protocMCP-1 and insulin on cholesterol efflux

ol number: SYSEC-KY-KS-2020-083] and was conducted according to the recommendations of the Declaration of Helsinki. All patients provided informed consent to participate in the study. Male patients with angiographically confirmed CAD (at least 50% obstruction of one or more major coronary arteries) were recruited from Sun Yat-sen Memorial Hospital of Sun Yat-sen University (Guangzhou, China). Among included male CAD patients, 28 kg/m^2^ > BMI ≥ 25 kg/m^2^ was defined as overweight and BMI ≥ 28 kg/m^2^ was obese. Healthy controls were recruited from Boji Medical Examination Center in Sun Yat-sen Memorial Hospital of Sun Yat-sen University (Guangzhou, China). A medical history and record reviewed CAD risk factors, including diabetes, hypertension, hypercholesterolemia history, drinking and cigarette smoking. Exclusion criteria included hypothyroidism, renal dysfunction, or hepatic failure. None of the patients participated in lipid-lowering therapy before blood collection. Blood was drawn after an overnight fast and collected in EDTA-coated tubes.

An Olympus AU-640 (Germany) was used for lipid analysis, and ELISA (BMS281TEN, Austria) was used for measuring MCP-1. The levels of HDL-C, HDL2-C, and HDL3-C were measured as in our previous study [17]. Briefly, HDL-C levels were measured in serum before separation. The serum samples (300 μl) were precipitated with heparin containing MnCl_2_ and dextran sulfate and separated by centrifugation at 10,000 rpm for 10 min. The amounts of HDL3-C in the supernatant were measured using homogeneous HDL-EX HDL-C assays (Denka Seiken, Tokyo, Japan). Levels of HDL2-C were derived from the following formula: HDL2-C=HDL-C-HDL3-C.

### Cell Culture

3T3-L1 preadipocytes were obtained from the American Type Culture Collection (ATCC, Cat # CL-173, RRID: CVCL_0123) and differentiated into adipocytes as described previously [19]. Briefly, preadipocytes were cultured in DMEM (Gibco/ Invitrogen, Carlsbad, CA, USA) containing 25 mM glucose and 10% CS (Gibco/ invitrogen) at 37 °C. Two days after the cultures became confluent (day 0), the cells were induced with DMEM containing 10% FBS (Gibco/invitrogen), 0.5 mM 1-methyl-3-isobutyl xanthine(Sigma-Aldrich, Louis, MO, USA), 1 mM dexamethasone (Sigma-Aldrich), and 1 μg/ml insulin(Sigma-Aldrich) (day 4). Four days later, the medium was changed to DMEM with 25 mM glucose, 10% FBS, and 1 μg/ml insulin for an additional four days (day 8). The medium was then changed to DMEM containing 25 mM glucose and 10% FBS until maturity (day 9–10). The process of 3T3-L1 differentiation were detected by microscope, as shown in Fig. S1. Prior to all experimental treatments, mature adipocytes were serum starved in DMEM containing 25 mM glucose and 0.2% bovine serum albumin (BSA) (Gibco/invitrogen) for 2 h at 37 °C. Different MCP-1 doses (0, 20, 40, and 80 ng/ml for 48 h) were added or for various durations (0, 24, 48, and 72 h at 40 ng/ml) were applied prior to the analyses.

### Cholesterol Efflux Assay

Fully differentiated adipocytes seeded in collagen-coated 24-well plates were starved for 6 h and labeled with [^3^H]-cholesterol (1 Ci/ml) (PerkinElmer Analytic Sciences, Boston, MA) for 24 h. Cellular cholesterol efflux was initiated by the addition of DMEM containing 0.2% BSA with 20 μg/ml human apoA-I or 50 μg/ml HDL2 with the indicated dose of MCP-1 for the indicated period of time in the presence or absence of insulin. After incubation, the radioactivity of the medium and cells was measured using a liquid scintillation counter. Cholesterol efflux was calculated as the quantity of labeled [^3^H]-cholesterol released into the medium divided by the total amount of label present.

### Real-Time PCR

Adipocytes were harvested in TRIzol reagent (Invitrogen, CA, USA), and RNA was isolated according to the manufacturer’s instructions. Then, 0.5 μg of total RNA was reverse-transcribed using a ReverTraAce-α-®kit (Toyobo, Osaka, JP). Real-time quantitative RT-PCR was performed using a Roche LightCycler 480 machine (Roche, Penzberg, Sweden) with SYBR® Premix-Ex TagTM (Takara, Dalian, China). The levels of ABCG1, ABCA1, and SR-BI gene expression were normalized to those of β-actin. The relative quantification of ABCG1, ABCA1, and SR-BI normalized to β-actin was calculated according to the 2^-(ΔΔCt)^ method.

### Western Blotting

3T3-L1 adipocytes were harvested after treatment with MCP-1 at various concentrations and for varying durations. The cells were washed twice with PBS at 4 °C and lysed in total protein extraction buffer (50 mM Tris-HCl, 150 mM NaCl, 2 mM EDTA, 0.1% SDS, 1% Triton X-100). The protein concentration was measured using a bicinchoninic acid (BCA) protein assay kit (Biocolor BioScience & Technology Company, Shanghai, China). Total proteins (30 μg/lane) were separated by 8% SDS-PAGE and transferred to polyvinylidene fluoride (PVDF) membranes. The blots were incubated in 5% BSA in TBST (100 mM Tris, pH 7.4, 0.9% sodium chloride, and 0.05% Tween-20) for 1 h and probed with primary antibodies overnight at 4 °C. The blots were washed, incubated in HRP-conjugated secondary antibodies, and visualized using chemiluminescence.

### Cell-Surface Protein Assays Using Biotinylation

After washing mature adipocytes five times with cold PBS (Life Technologies, Carlsbad, CA, USA) containing 0.1 mM CaCl2 and 1 mM MgCl2 (PBS-CM), the cells were labeled twice with 0.5 mg/ml EZ-Link Sulfo-NHS-SS-Biotin (Pierce Chemical Co., Rockford, IL) on ice for 20 min. Non-reacted biotin was quenched and removed by washing with Tris-buffered saline-CM for 5 min. The cells were lysed with RIPA buffer on ice for 30 min and centrifuged at 4 °C and 12,000×*g* for 30 min. After quantification, the supernatants of the cell lysates were incubated with NeutrAvidin Agarose Resin overnight at 4 °C and pelleted by centrifugation. The pellets were collected with SDS loading buffer and analyzed using western blotting, as described above.

### Confocal Microscopy

3T3-L1 adipocytes were plated on 12-mm glass coverslips and cultured as described above. The cells were rinsed three times (5 min each) in PBS, fixed with 4% paraformaldehyde for 20 min at room temperature (RT), and fixed with cold 100% methanol for 5 min at −20 °C. The cells were rinsed an additional three times in PBS and blocked for 1 h at RT in PBS containing 10% donkey serum (Jackson Immunoresearch Inc., Baltimore, PA, USA) and 5% BSA. The adipocytes were incubated at 4 °C overnight with a 1:25 dilution of mouse anti-mouse ABCA1 antibody, a 1:200 dilution of rabbit anti-mouse ABCG1 antibody and a 1:50 dilution of goat anti-mouse SR-BI antibody (Novus Biologicals, CO, USA) in blocking buffer. Subsequently, the cells were incubated with the following fluorescent secondary antibodies: Alexa Fluor® 546 goat anti-mouse IgG, Alexa Fluor® 488 donkey anti-rabbit, and Alexa Fluor® 633 donkey anti-goat IgG for ABCA1, ABCG1, and SR-B1, respectively. After three rinses in PBS, nuclei were stained with DAPI (1 μg/ml) (Invitrogen, Carlsbad, CA, USA) for 5 min. The cells were visualized by confocal microscopy.

### Data and Statistical Analysis

The data were statistically analyzed using IBM SPSS 26.0 software, and the continuous data are expressed as the means ± SD (for normally distributed variables) or medians with interquartile ranges (for non-normally distributed variables). Categorical variables were expressed as case numbers (percentages). To compare the normally distributed variables, the statistical significance of differences was determined using Student’s *t* test, non-parametric test, or one-way analysis if variable followed by Bonferroni’s post hoc test, as appropriate. Categorical variables were compared using the *χ*^*2*^ test. Correlations between plasma MCP-1concentration and the variables were analyzed using Pearson correlation coefficients. *P* < 0.05 was considered statistically significant.

## Results

### MCP-1 Is Negatively Correlated with HDL2-C and apoA-I Levels in Male CAD Patients with Overweight and Obesity

**Fig. 1:**
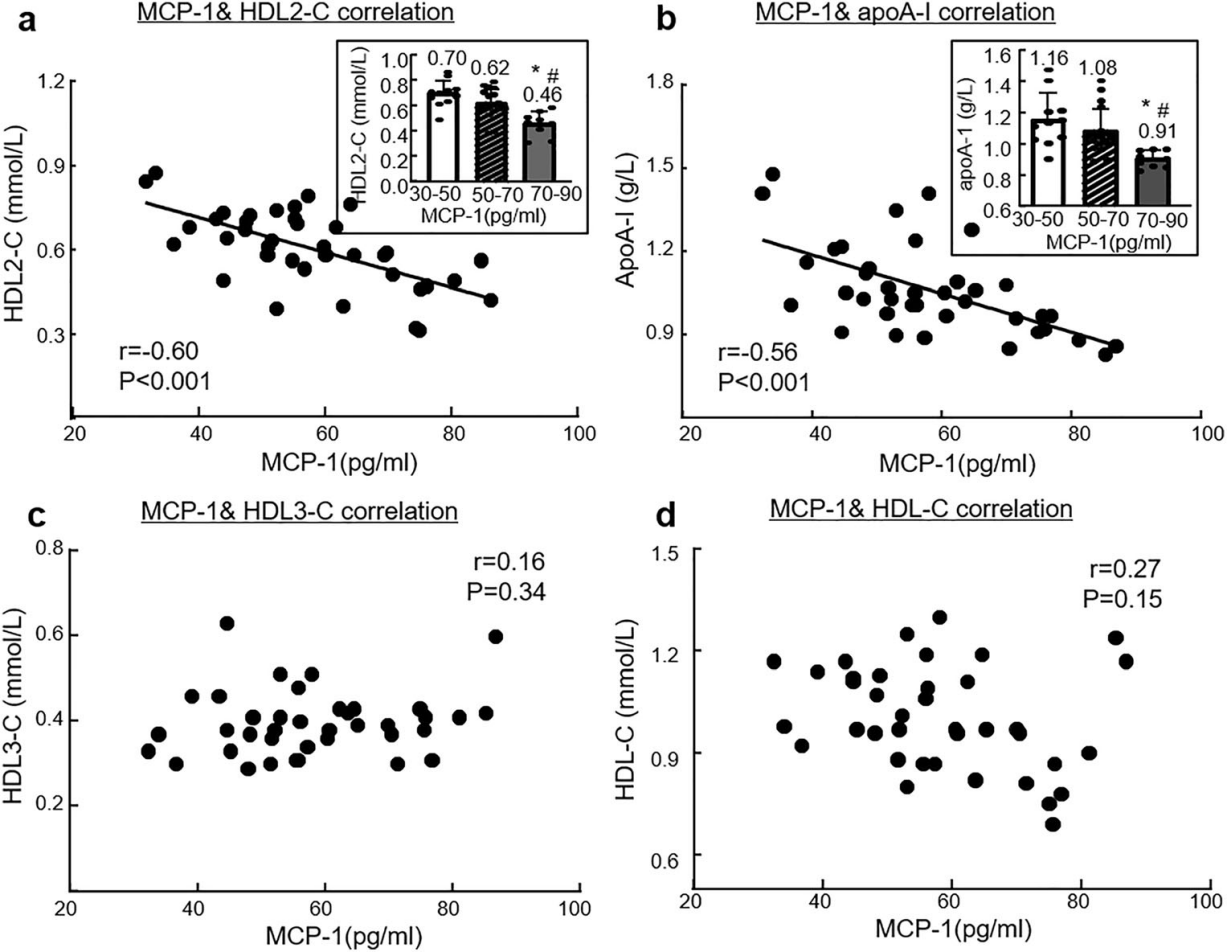
MCP-1 was negatively corelated with HDL2-C and apoA-I in male CAD patients with overweight and obesity. Plasma of fasted male CAD patients with overweight and obesity (*n* = 38) was analyzed for HDL2-C, HDL3-C, HDL-C, apoA-I, and MCP-1. Pearson correlation was performed. Each data point represents 1 patient. Inner bar graph shows HDL2-C or apoA-I protein levels in grouped MCP-1 CAD patients with overweight and obesity. **(a)** Correlation of HDL2-C vs plasma levels of MCP-1. **(b)** Correlation of apoA-I vs plasma levels of MCP-1. **(c)** Correlation of HDL3-C vs plasma levels of MCP-1. **(d)** Correlation of HDL-C vs plasma levels of MCP-1. Note that both HDL2-C and apoA-I are negatively correlated with MCP-1 levels. *P < 0.05 vs 30–50 pg/ml MCP-1 group, ^#^*P* < 0.05 vs 50–70 pg/ml MCP-1 group

The demographic and clinical characteristic of male CAD patients and healthy controls were shown as Table S1. Compared with the healthy controls, CAD patients had higher MCP-1 levels and lower HDL-C, HDL2-C, HDL3-C, and apoA-I levels (*p* < 0.05, Table 1). Also, CAD patients had higher BMI and lower HDL3-C/HDL2-C than healthy controls (Table 1). Ninety-eight male CAD patients were divided into three groups, based on BMI, as normal (18 to 24 kg/m^2^), overweight (25 to 28 kg/m^2^), and obesity (≥28 kg/m^2^) (Table 2). HDL-C in the three groups were not different. In contrast, HDL2-C and apoA-I levels in CAD patients with obesity were lower than those of the normal BMI group (0.55 ± 0.13 mmol/L vs 0.67 ± 0.11 mmol/L, 0.98 ± 0.12 g/L vs 1.12 ± 0.13 g/L, respectively), while MCP-1 levels in CAD patients with obesity were the highest among the three groups (normal 53.02 ± 10.76 mmol/L, overweight 56.21 ± 13.12 mmol/L, obesity 62.83 ± 15.63 mmol/L). Furthermore, increased MCP-1 levels had a significant, negative correlation with HDL2-C and apoA-I concentration (*r* = −0.60, *p* < 0.001; *r* = −0.56, *p* < 0.001, Fig.1a and b) but no correlation with HDL3-C and HDL-C (*r* = 0.16, *p* = 0.34; *r* = 0.27, *p* = 0.15, Fig. 1c and d).

**Table 1:** Comparison of parameters associated with MCP-1 levels and HDL2-C in male CAD patients and healthy controls

	Healthy controls (*n* = 136)	CAD patients (*n* = 98)	*t/Z*	*P* value
BMI(Kg/m^2^)	23.13 ± 1.93	23.82 ± 2.83	-2.193^a^	0.029*
MCP-1(pg/ml)	40.76 ± 12.27	55.00 ± 12.31	-8.745^a^	<0.001*
HDL-C(mmol/L)	1.26 ± 0.20	1.04 ± 0.14	9.408^a^	<0.001*
HDL2-C(mmol/L)	0.76 ± 0.12	0.65 ± 0.12	6.972^a^	<0.001*
HDL3-C(mmol/L)	0.50 ± 0.09	0.39 ± 0.06	9.987^a^	<0.001*
apoA-I(g/L)	1.29 ± 0.16	1.11 ± 0.15	9.279^a^	<0.001*
HDL3-C/HDL2-C	0.68(0.64, 0.71)	0.62(0.52,0.67)	−5.078^b^	<0.001*

Continuous variables were expressed as the means ± SD (for normally distributed variables) or medians with interquartile ranges (for non-normally distributed variables). BMI, body mass index; CAD, coronary artery disease; MCP-1, monocyte chemoattractant protein-1; HDL-C, high density lipoprotein cholesterol; HDL-2C, high density lipoprotein 2 cholesterol; HDL3-C, high density lipoprotein 3 cholesterol; apoA-I, apolipoprotein A-I. ^a^ represents the *t* statistic; ^b^ represents the *Z* statistic. *P < 0.05 as significance

**Table 2:** Plasma levels of MCP-1 and HDL2-C in male CAD patients with overweight and obesity

Group	N	BMI (Kg/m^2^)	MCP-1 (pg/ml)	HDL-C (mmol/L)	HDL2-C (mmol/L)	HDL3-C (mmol/L)	apoA-I (g/L)
Normal	60	21.88 ± 1.15	53.02 ± 10.76	1.06 ± 0.13	0.67 ± 0.11	0.39 ± 0.05	1.12 ± 0.13
Overweight	27	25.92 ± 1.00^*^	56.21 ± 13.12	1.01 ± 0.16	0.63 ± 0.14	0.38 ± 0.06	1.09 ± 0.17
Obesity	11	29.22 ± 1.01^§^	62.83 ± 15.63^*^	0.99 ± 0.13	0.55 ± 0.13^*^	0.43 ± 0.10^*§^	0.98 ± 0.12^*§^

Continuous variables were expressed as the means ± SD (for normally distributed variables) or medians with interquartile ranges (for non-normally distributed variables). BMI, body mass index; CAD, coronary artery disease; MCP-1, monocyte chemoattractant protein-1; HDL-C, high density lipoprotein cholesterol; HDL-2C, high density lipoprotein 2 cholesterol; HDL3-C, high density lipoprotein 3 cholesterol; apoA-I, apolipoprotein A-I. *P < 0.05 vs normal group, ^§^*P* < 0.05 vs overweight group

### MCP-1 Reduces Cholesterol Efflux to Large HDL2 Particles in Differentiated 3 T3-L1 Adipocytes

We found increased MCP-1 is negatively correlated with decreased HDL2-C and apoA-I levels in CAD patients with overweight and obesity. We speculated MCP-1 impaired large HDL2 particles induced cholesterol metabolism of adipocytes in obesity (apoA-I was as a positive control).

**Fig. 2:**
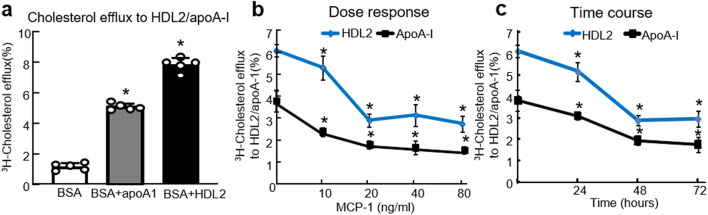
MCP-1 suppressed [^3^H]-cholesterol efflux to large HDL2 particles in differentiated 3T3-L1 adipocytes. **(a)** basal cholesterol efflux to large HDL2 particles and apoA-I in adipocytes. Fully differentiated adipocytes seeded on collagen-coated 24-well plates were starved for 6 h and labeled with [^3^H]-cholesterol (1 Ci/ml) for 24 h. Efflux was initiated by BSA alone and BSA plus 20 μg/ml ApoA1 or 50 μg/ml HDL2 for 2 h. ApoA-I was the major protein of HDL particles and as the positive control. The radioactivity of the medium and cells was measured with a liquid scintillation counter. The cholesterol efflux was expressed as the percentage of counts in the medium relative to the total counts for the medium and cells together. **(b)** Dose response of MCP-1 on cholesterol efflux to HDL2/apoA-1. Cells were initiated by 50 μg/ml human HDL2 or 20 μg/ml apoA-1 for 2 h after treating with increasing doses (0, 10, 20, 40, and 80 ng/ml) of MCP-1. The final cholesterol efflux was calculated as the percentage of total ^3^H cholesterol released into the medium after subtraction of the values obtained in the absence of HDL2/apoA-1. Other procedures are the same as **(a). (c)** Time course of MCP-1 on cholesterol efflux to HDL2/ApoA1. Cells were incubated with 50 μg/ml human HDL2 or 20 μg/ml apoA-I for 2 h after treating with 40 ng/ml MCP-1 for indicate times (0, 24, 48, and 72 h). Others were the same as **(a)**. The results were expressed as mean ± SD (**(a–c)** *n* = 3). * *P* < 0.05 compared with the untreated cells

Next, we examined the effect of MCP-1 on cholesterol efflux to large HDL2 particles in differentiated 3T3- L1 adipocytes. First, 3T3-L1 adipocytes had a basal rate of [^3^H]-cholesterol efflux to BSA at 1.17%. Also, HDL2 and apoA-I increased the [^3^H]-cholesterol efflux from 3T3-L1 adipocytes to 7.85% and 5.09%, respectively (Fig. 2a). In a dose-dependent experiment, we treated 3T3-L1 cells for 48 h with increasing doses of MCP-1 (0–80 ng/ml, Fig. 2b). MCP-1 significantly reduced cholesterol efflux to large HDL2 particles and apoA-I by 51.8% (20 ng/ml) and 55.4% (40 ng/ml), respectively, compared with the untreated group. To investigate the time-course responses of cholesterol efflux to HDL2 and apoA-I, we treated 3T3-L1 adipocytes with 40 ng/ml MCP-1 for increasing durations (0–72 h). Also, MCP-1 treatment resulted in the largest reduction of 52.8% in cholesterol efflux to HDL2 at 48 h compared with untreated cells. In addition, cholesterol efflux to apoA-I was significantly reduced from the control level at 72 h (Fig. 2c). Therefore, cholesterol efflux to HDL2 and apoA-I is decreased by MCP-1 in both a dose- and time-dependent manner. Furthermore, MCP-1 reduced the cholesterol efflux to HDL2 and HDL3 by 3.1% and 3.1%, but the decrease rate was 51.7% (3.1/6.0*100%) and 37.3% (3.1/8.3*100%), respectively. MCP-1 suppressed cholesterol efflux to HDL2 much more efficiently than HDL3 (Fig. S2).

### MCP-1 Decreased mRNA, Total Protein, and Cell Surface Protein Expression of ABCA1, ABCG1, and SR-BI in Adipocytes

**Fig. 3:**
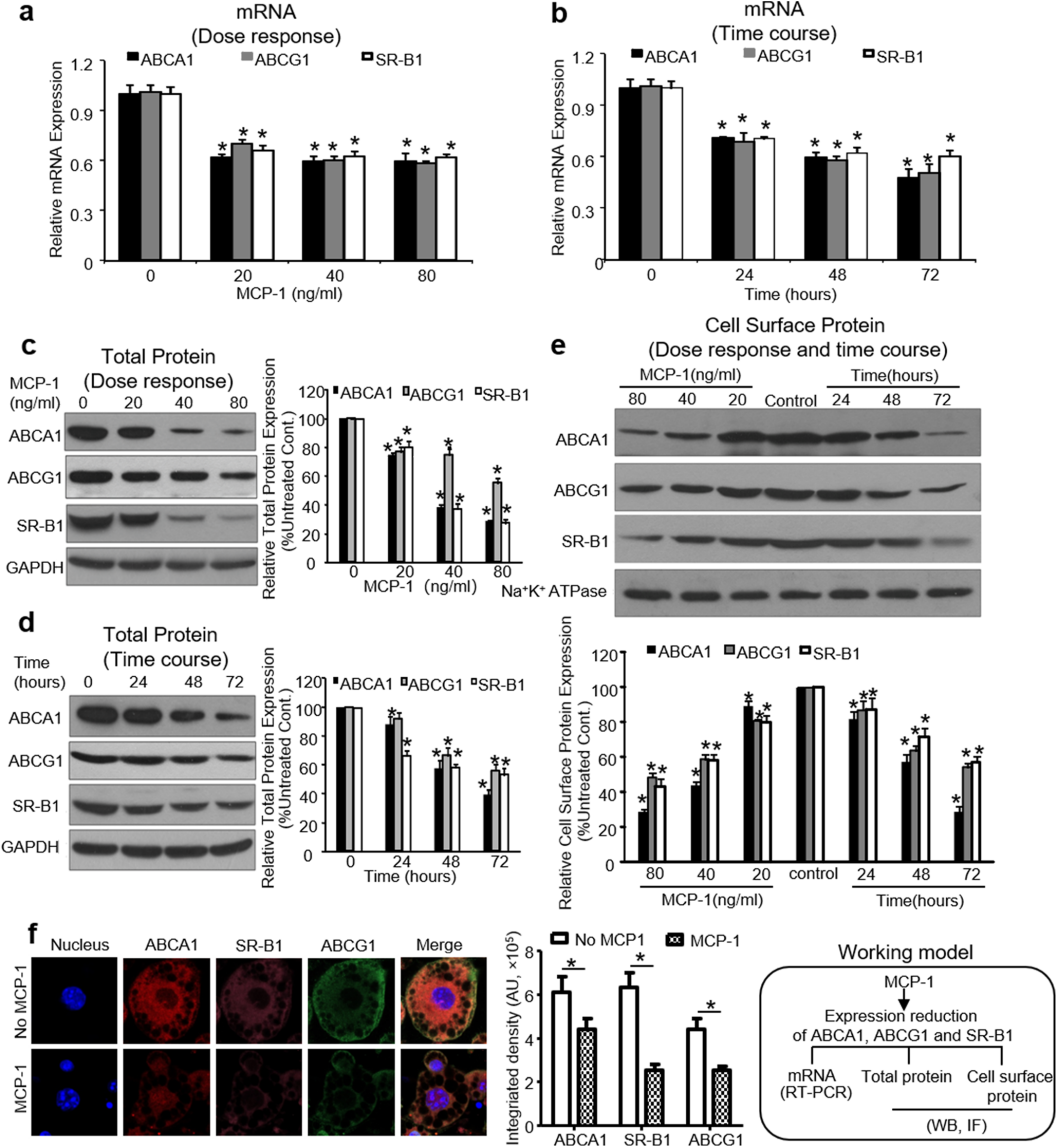
MCP-1 inhibited the mRNA, total, and cell-surface protein expression of ABCA1, ABCG1, and SR-B1 in differentiated 3T3-L1 adipocytes. Differentiated 3T3-L1 adipocytes were treated with either increasing concentrations of MCP-1 (0–80 ng/ml) for 48 h or with MCP-1 at 40 ng/ml for increasing times (0, 24, 48, 72 h). **(a)** Dose response of MCP-1 on ABCA1, ABCG1, and SR-B1 mRNA expression. **(b)** Time course of MCP-1 on ABCA1, ABCG1, and SR-B1 mRNA expression. Total proteins and cell surface proteins were extracted from the cultured cells, and the protein expressions of ABCA1, ABCG1, and SR-B1 were detected as described in “Materials and Methods.” The quantitative analysis of proteins was performed using Image-J software. **(c)** Dose response of MCP-1 on the total protein expression of ABCA1, ABCG1, and SR-B1. **(d)** Time course of MCP-1 on the total protein expression of ABCA1, ABCG1, and SR-B1. **(e)** Dose response and time course of MCP-1 on the cell surface protein expression of ABCA1, ABCG1, and SR-B1. **(f)** The effect of MCP-1 on the expression of ABCA1, ABCG1, and SR-B1 by confocal microscopy. Differentiated 3T3-L1 adipocytes grown on glass cover slips were serum-starved for 6 h, followed by incubation in serum-free medium in the absence or presence of MCP-1 (40 ng/ml) for 48 h. ABCA1, ABCG1, and SR-BI were labeled with Alexa 546 (red), Alexa 488 (green), and Alexa 633 (pink), respectively. The protein expression of ABCA1, ABCG1, and SR-BI was analyzed using confocal microscopy (LSM780) (×63), as described in “Materials and Methods.” (**(a–f)**, n = 3). *P < 0.05 compared with untreated cells

ABCA1, ABCG1, and SR-BI play important roles in cholesterol efflux and are crucial for regulating cellular cholesterol homeostasis. Thus, the effect of MCP-1 on the mRNA and protein expression levels of these receptors was performed in adipocytes. Differentiated 3T3-L1 adipocytes were treated by MCP-1 in the same manner as for the cholesterol efflux study. 40 ng/ml MCP-1 repressed the mRNA expression of ABCA1, ABCG1, SR-BI by approximately 40.7%, 40.0%, and 37.9%, respectively (Fig. 3a). Figure 3b shows the time-dependent response of mRNA expression to 40 ng/ml MCP-1, and the ABCA1, ABCG1, and SR-BI mRNA expression were declined by 52.6%, 49.3%, and 40.1% at 72 h, respectively, compared with the levels in untreated cells.

To investigate whether MCP-1 could alter the total protein levels of ABCA1, ABCG1, or SR-BI, we treated differentiated 3T3-L1 adipocytes in the same manner as for the mRNA expression experiment. It is shown that 40 ng/ml MCP-1 decreased the protein levels of ABCA1, ABCG1, and SR-BI to 40%, 78%, and 39%, respectively, compared with the untreated group (Fig. 3c). It revealed the time-course responses of ABCA1, ABCG1, and SR-BI total protein expression to 40 ng/ml MCP-1, the inhibition of ABCA1, ABCG1, and SR-BI protein expression was evident at 48 h mostly (40%, 30%, and 38%, respectively) compared with the untreated cells (Fig. 3d).

Studies suggested that the redistribution of ABCA1, ABCG1, and SR-BI can be stimulated and that such redistribution toward the cell membrane would in turn affect cholesterol efflux. It was unclear whether the cell surface expression of ABCA1, ABCG1, and SR-BI was similar to the expression of total protein in the presence of MCP-1. We thus next quantified the changes in cell surface protein expression induced by MCP-1 by directly measuring the number of cell surface receptors by cell surface protein biotinylation. The cell surface expression levels of ABCA1, ABCG1, and SR-BI were reduced in a dose-dependent and time-dependent manner after treatment with 40 ng/ml MCP-1 for 48 h (Fig. 3e). To confirm the reduction in receptor expression in 3T3-L1 adipocytes treated with MCP-1, the cells were examined under confocal microscopy. Adipocytes were equilibrated for 6 h and incubated in serum-free medium in the presence or absence of MCP-1 (40 ng/ml) for 48 h, and both the cytoplasmic and cell surface expression of ABCA1, ABCG1, and SR-BI proteins was inhibited by MCP-1 (Fig. 3f), which was in line with the changes detected by western blotting.

### Insulin Increased the MCP-1-Suppressed Cholesterol Efflux to Large HDL2 Particles in an Akt Phosphorylation-Dependent Manner

The impact of insulin on MCP-1-suppressed cholesterol efflux to large HDL2 particles remains unknown. Moreover, other studies demonstrated the inhibition of cholesterol efflux by MCP-1 involves PI3K/Akt. Thus, we speculated whether insulin could reverse the effects of MCP-1 through Akt phosphorylation.

**Fig. 4:**
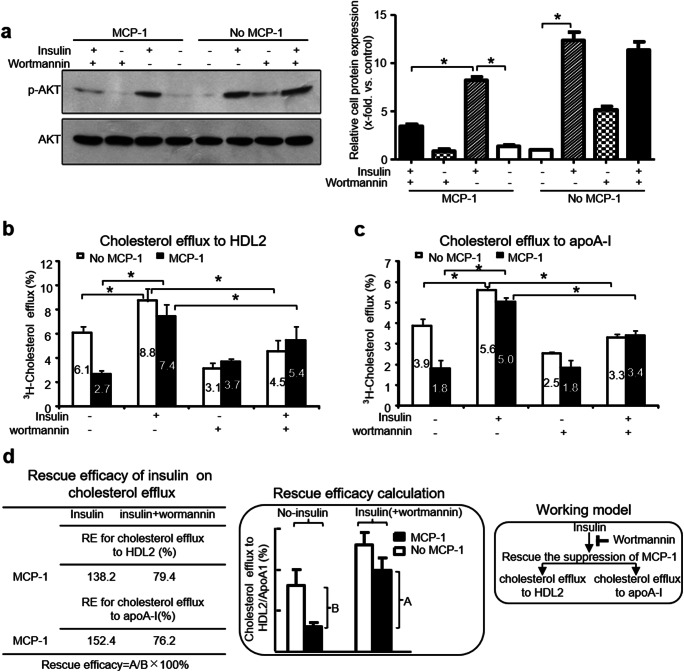
Insulin restored cholesterol efflux to large HDL2 particles suppressed by MCP-1 in an Akt phosphorylation-dependent manner. Starved matured 3T3-L1 adipocytes were pretreated with a PI3K inhibitor (wortmannin, 100 nM) and then incubated with insulin (100 nM) in the presence of MCP-1 (40 ng/ml) for 48 h. **(a)** Insulin induced Akt phosphorylation. Total proteins were extracted from the cultured cells, and the protein levels were analyzed using western blotting, as described in “Materials and Methods.” The relative expression of p-Akt is expressed as the x-fold vs control in ratio of p-Akt to total Akt. **(b)** Insulin reversed the MCP-1 suppression on cholesterol efflux to large HDL2 particles in an Akt-dependent manner. ^3^H-cholesterol efflux to HDL2 was determined as described in “Materials and Methods.” **(c)** Insulin reversed the MCP-1 suppression on cholesterol efflux to ApoA-I in an Akt-dependent manner. ^3^H-cholesterol efflux to ApoA-I was determined as described in “Materials and Methods.” **(d)** Rescue efficacy (RE) of insulin on MCP-1-induced decreased in cholesterol efflux (RE = A/B × 100%). (**(a–d)**: n = 3) *P < 0.05

First, we explored whether insulin could alter the Akt phosphorylation in adipocytes. We assessed Akt phosphorylation with an antibody specific to Ser 473-phosphorylated Akt. Mature 3T3-L1 adipocytes were treated with insulin and a PI3K inhibitor (100 nM wortmannin) in the presence of MCP-1. Results showed insulin increased the expression of p-Akt in the presence of MCP-1. Moreover, p-Akt was decreased by wortmannin. These results suggested that insulin activated PI3K/Akt in the presence of MCP-1(Fig. 4a).

Next, we determined [^3^H]-cholesterol efflux to HDL2 in adipocytes treated with insulin (100 nM) for 45 min in the presence of MCP-1 (40 ng/ml, 48 h). In the presence of MCP-1, insulin increased the suppression of cholesterol efflux to large HDL2 particles and apoA-I (177.1% and 176.7%, respectively), and this effect was blocked by wortmannin (Fig. 4b and c). Furthermore, we established a parameter of rescue efficacy (RE) to quantitatively address the capacity of insulin on rescuing cholesterol efflux (Fig. 4d). The rescue efficacy of insulin was 138.2% and 152.4% for HDL2 and apoA-I, respectively. Moreover, rescue efficacy (RE) of insulin on cholesterol efflux was suppressed by wortmannin. Therefore, insulin rescued the MCP-1-suppressed cholesterol efflux to HDL2 in an Akt phosphorylation-dependent manner.

### Insulin Reverses the MCP-1-Mediated Suppression of ABCA1, ABCG1, and SR-BI mRNA Expression and Protein Expression Via the PI3K/Akt Pathway

**Fig. 5:**
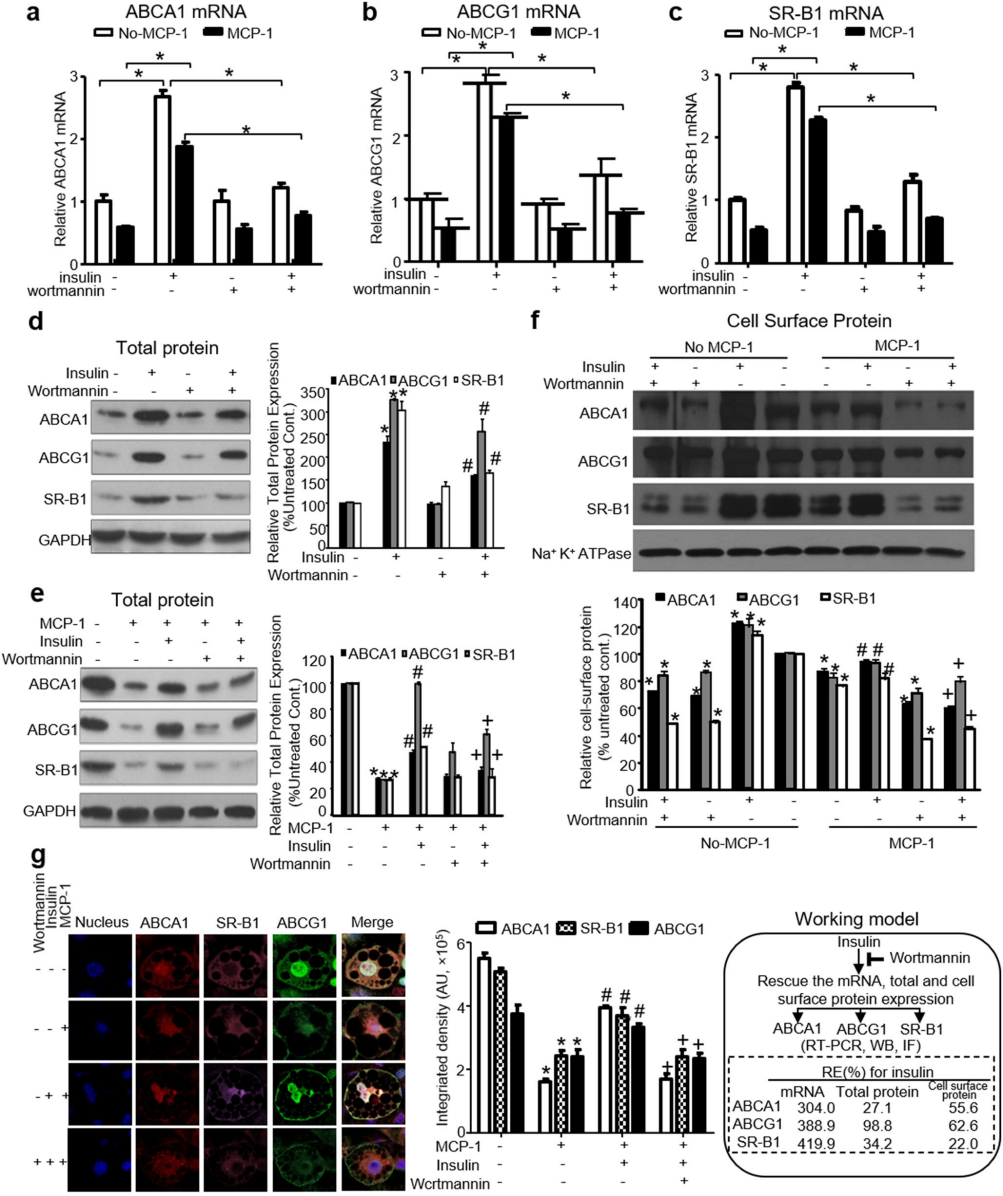
Insulin rescues MCP-1-mediated mRNA, total and surface protein suppression of ABCA1, ABCG1, and SR-B1 via PI3K/Akt activation in differentiated 3T3-L1 adipocytes. Cells were serum-starved for 6 h, followed by pretreatment with (+) or without (−) the PI3K inhibitor wortmannin for 45 min. The cells were then incubated with (+) or without (−) insulin (100 nM) and MCP-1 (40 ng/ml). **(a–c)** Insulin rescues MCP-1 mediated mRNA suppression of ABCA1, ABCG1and SR-B1. **(d, e)** Insulin rescues the total protein expression of the three receptors. **(f)** Insulin rescues the surface protein expression of the three receptors. Cell surface receptor levels and Na^+^/K^+^ ATPases were directly extracted using cell surface biotinylation and then measured using western blotting, as described in “Materials and Methods.” **(g)** Insulin rescues the expression of the three receptors by confocal microscopy (×63). ABCA1, SR-BI, and ABCG1 labeled with Alexa 546 (red), Alexa 633 (pink), and Alexa 488 (green), respectively, were detected by confocal microscopy as describe in “Materials and Methods.” Note that insulin reversed the MCP-1-mediated decreases in ABCA1, ABCG1, and SR-BI mRNA and protein levels, and this correction was inhibited by wortmannin. (**(a–g)**: n = 3). *P < 0.05 compared with untreated cells; ^#^*P* < 0.05 compared with MCP-1- or insulin-treated cells; ^**+**^*P* < 0.05 compared with MCP-1 or insulin-treated cells

We investigated whether insulin could increase the mRNA and protein expression of ABCA1, ABCG1, and SR-BI suppressed by MCP-1 through the PI3K/Akt pathway. Cells were pretreated with or without MCP-1 (40 ng/ml) for 48 h and then treated with insulin for 48 h. To observe PI3K/Akt regulation, 3T3-L1 adipocytes were pretreated with wortmannin before insulin added with or without MCP-1. Insulin could reverse the downregulation of ABCA1, ABCG1, and SR-BI mRNA expressions by MCP-1, and this effect could be blocked by wortmannin (PI3K inhibitor) (Fig. 5a-c).

Next, we determined whether insulin could regulate the total protein expression of ABCA1, ABCG1, and SR-BI inhibited by MCP-1. Interestingly, the suppression of the total protein expression of these three receptors by MCP-1 was reversed by insulin, and this rescue could also be blocked by wortmannin (Fig. 5d and e).

We further investigated the cell surface expression of ABCA1, ABCG1, and SR-BI regulated by insulin or PI3K activity. Membrane protein levels were measured using cell surface biotinylation, which was performed after insulin and MCP-1 treatment with or without PI3K inhibition (wortmannin). Strikingly, with MCP-1 treatment, insulin reversed the reduction of these three membrane proteins, and this effect was also blocked by wortmannin (Fig. 5f).

Furthermore, confocal microscopy was performed to observe the changes of these three transporters in differentiated 3T3-L1 adipocytes after insulin treatment as well as the PI3K/Akt regulation of this process. Cells were equilibrated for 6 h, incubated in serum-free medium, and treated as above. ABCA1, ABCG1, and SR-BI levels were reduced throughout the cytoplasm and at the cell surface by MCP-1 treatment. Additionally, insulin rescued both the cytoplasmic and the cell surface expression of these three transporters suppressed by MCP-1, and this effect could also be blocked by wortmannin (Fig. 5g). These findings were in accordance with the changes in total and cell surface protein changes detected by western blotting. Also, RE of insulin on mRNA, total protein and cell surface protein expression of ABCA1, ABCG1, and SR-B1 was shown in the working model of Fig. 5.

## Discussion

**Fig. 6:**
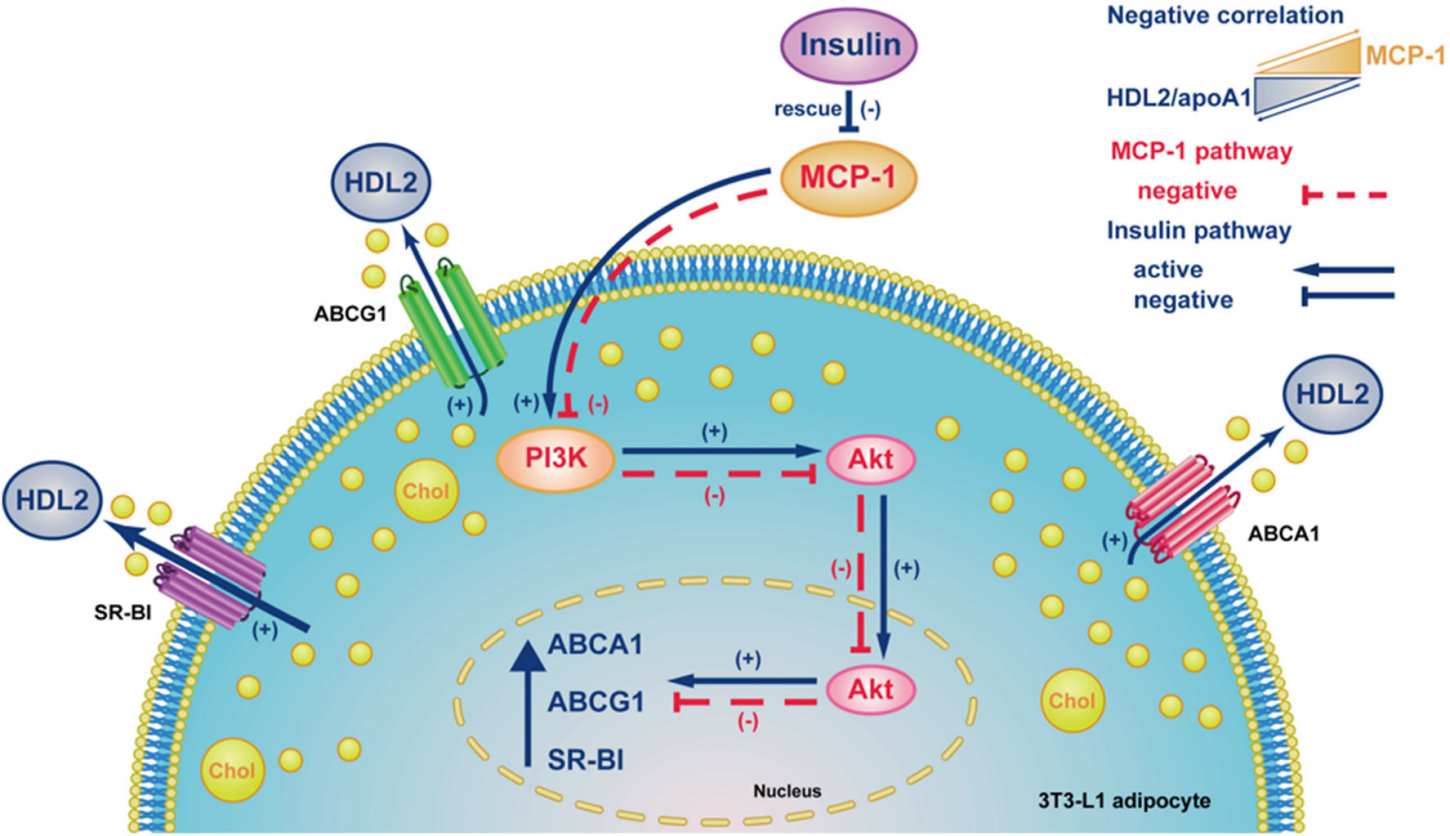
Proposed model of the effect of Insulin on MCP-1-mediated suppression of cholesterol efflux to large HDL2 particles and regulatory mechanism in 3T3-L1 adipocytes. Increased MCP-1 level has a significant, negative correlation with HDL2-C in CAD patients with obesity and overweight. MCP-1 decreases the expression of ABCA1, ABCG1, and SR-B1, leading to the reduction of cholesterol efflux. Insulin rescues the expression of the three receptors via PI3K/Akt activation and restores the cholesterol efflux to HDL2. (+), Activation; (−), Inhibition

Our data suggest that increased MCP-1 level has a significant, negative correlation with HDL2-C in CAD patients with obesity and overweight. Insulin rescues the MCP-1-suppressed cholesterol efflux to large HDL2 particles by increasing ABCA1, ABCG1, and SR-BI expression at both transcriptional and translational levels via PI3K/Akt activation (Fig. 6). Our findings provide the first evidence that insulin restores the suppression of cholesterol efflux to HDL2 by inflammatory response in adipocytes.

Adipose tissue is the largest pool of cholesterol in the body [20], and intracellular cholesterol imbalance in adipocytes has been suggested to play an important role in adipose tissue dysfunction [9]. Adipose tissue dysfunction is the main feature of obesity, and also promotes cardiovascular disease [21]. Because the ability of adipocytes to synthesize and catabolize cholesterol is very limited [9], the cholesterol content of adipose tissue depends on the balance between cholesterol efflux and influx. Therefore, the rate of cholesterol efflux affects the cholesterol content of adipose tissue to a greater degree compared with other tissues such as the liver [22]. Adipocyte inflammation downregulates transporters, including ABCA1, ABCG1, and SR-BI and impairs adipocyte cholesterol efflux to HDL [23]. Moreover, a reduction in inflammation is associated with improvement in cholesterol efflux capacity [24, 25]. Studies find that MCP-1 in adipose tissues plays an important role in obesity [5, 26]. Our data also shows that MCP-1 impairs cholesterol efflux from adipocytes. The anti-inflammatory effect of insulin has been documented in blood cells, endothelial cells, and adipose tissue [27, 28], and the ability of insulin to suppress the induction of the pro-inflammatory chemokine MCP-1 was shown in human aortic endothelial cells [29]. Our previous study [17] showed that MCP-1 inhibited the cholesterol efflux from human coronary artery endothelial cells, which was in line with this study.

The HDL subfractions, HDL2 and HDL3, are particles with unique size and density that may have distinct cholesterol efflux capacities [30]. Our data showed that HDL2, a large particle subfraction, possessed a higher cholesterol efflux rate than apoA-I (Fig. 2a). It is speculated that HDL2 has more proteins related with cholesterol efflux, such as apoA-IV, except for apoA-I [31]. Our findings showed that in CAD patients with overweight and obesity, HDL2-C was decreased, and MCP-1 concentration was negatively corelated with HDL2-C level (Fig. 1a). We considered MCP-1 may inhibit reverse cholesterol transport of large HDL2 particles.

Different potential cellular cholesterol efflux pathways have been described, including diffusional efflux and ABCA1-, ABCG1-, and SR-BI-mediated cholesterol efflux pathways [32, 33]. 3 T3-L1 adipocytes express several important cholesterol transporters such as ABCA1 and ABCG1 [22, 34], but controversies exist regarding cholesterol efflux from adipocytes. SR-BI is expressed in differentiated 3 T3-L1 adipocytes [35], but little is known about the function of SR-BI in adipose tissue, where it contributes to cholesterol efflux. Early studies in 3 T3-L1-derived adipocytes have shown that despite a strong induction of ABCA1 mRNA during differentiation, cholesterol efflux through the ABCA1 pathway remains limited [34]. In addition, cholesterol content in adipocytes isolated from a patient with Tangier disease is normal [36]. However, many studies indicate that ABCA1 plays a major role in cholesterol efflux from adipose tissue [15, 37]. Gonadal adipose tissue (GAT) lacking ABCA1 has reduced cholesterol efflux and increased cholesterol stores [38], and mice specifically lacking ABCA1 in adipocytes (ABCA1^-ad/−ad^) show increased cholesterol stores in adipose tissue, enlarged fat pads, impaired glucose tolerance, and lower insulin sensitivity [15]. Acute phase serum amyloid A can mediate ABCA1-dependent cholesterol efflux, whereas low concentrations of ox-LDL are capable of increasing cholesterol efflux by stimulating the ABCA1 pathway [39].

Previous studies demonstrate that ABCG1 is not highly expressed in adipocytes [14] but can be induced by a synthetic liver X receptor (LXR) agonist [40]. A study using single-receptor-deficient mice indicates that both ABCA1 and SR-BI but not ABCG1 are implicated in adipocyte cholesterol efflux [23]. However, other studies have indicated that ABCG1 may play an important role in cholesterol homeostasis in adipose tissue by mediating cholesterol efflux to HDL [41]. A study showed that the adipose tissue level of ABCG1 was significantly increased in obese mice and that the level was further elevated markedly with caloric restriction, which demands more cholesterol efflux [22]. Moreover, another study demonstrated that the silencing of ABCG1 expression in differentiated 3 T3-L1 adipocytes is accompanied by a significant reduction in cholesterol efflux to HDL [42]. Studies of double-deficient mice show that ABCA1 and ABCG1 operate synergistically to remove cellular cholesterol in several cell types and that ABCA1 may substitute for ABCG1 activity in cholesterol efflux [43]. Thus, it is possible that ABCG1 is operational in adipocytes during demand lipolysis. In the present study, we found that MCP-1 reduced cholesterol efflux to large HDL particles mainly by inhibiting SR-BI, ABCG1, and ABCA1 in 3T3-L1 adipocytes. These data suggest that ABCA1, ABCG1, and SR-BI play a central role in cholesterol efflux to HDL2 in adipocytes. However, the interaction of ABCA1, ABCG1, and SR-BI in adipocytes in mediating cholesterol efflux needs further investigation.

Large-cohort studies examining the effects of insulin on lipids have demonstrated reductions in total cholesterol (TC), LDL-C, and TGs and increases in HDL-C [44]. Therefore, insulin is closely related to lipid metabolism. Insulin has also been reported to influence cholesterol removal from different cells, but the results have been controversial. Some studies found that insulin counteracts cholesterol removal from several cell types, such as human macrophages [19, 45] and skin fibroblasts [46] in vitro, and further studies into the mechanisms are ongoing. Park et al. found that insulin decreased ABCA1 expression by more than 80%, which resulted in decreased cholesterol efflux through the PI3K/Akt pathway in macrophages [47]. Sealls et al. indicate that hyperinsulinemia results in elevated endosomal membrane ABCA1 and diminished plasma membrane ABCA1, which substantially increases cellular cholesterol [48]. Yamashita et al. report that insulin suppresses HDL-mediated cholesterol efflux from THP-1-derived macrophages through the inhibition of nCEH and ABCG1 expression [18]. We suspect that the effects of insulin on cholesterol transporters and cholesterol efflux are different in different cell types. However, little is known about the effect of insulin on cholesterol efflux in adipocytes. Our present data indicate that insulin can significantly alleviate the inhibition of cholesterol efflux to HDL2 caused by MCP-1 through increasing the suppressed expression of ABCA1, ABCG1, and SR-BI at both transcriptional and post-transcriptional levels in adipocytes. Moreover, these effects of insulin were suppressed by PI3K inhibition, and the repressed phosphorylation of Akt by MCP-1 was improved by insulin. Therefore, the PI3K/Akt pathway participates in the regulation of cholesterol transporters by insulin. Thus, we demonstrate that insulin improves the intracellular cholesterol imbalance by regulating the expression of cholesterol transporters in adipocytes. These results are analogous to another study [12] that demonstrated that the reduced intracellular accumulation of cholesterol in macrophages from CEH transgenic mice will attenuate the expression of pro-inflammatory mediators and improve insulin sensitivity.

Cholesterol efflux is the first step of reverse cholesterol transport (RCT). Our study establishes a link between MCP-1, HDL2, and adipocyte RCT and provides the first experimental evidence for the PI3K/Akt-dependent mechanism of cholesterol imbalance mediated by MCP-1 at the molecular and cellular levels using adipocytes relevant to obesity. Furthermore, insulin blocking MCP-1 appears to be a viable strategy for improving adipocyte RCT and is currently being pursued in clinical trials.

In conclusion, insulin rescues the cholesterol efflux to large HDL2 particles that are suppressed by MCP-1 through the upregulation of ABCA1, ABCG1, and SR-BI via the PI3K/Akt pathway in 3T3-L1 adipocytes. These data provide evidence that insulin can improve the MCP-1-induced adipocyte cholesterol imbalance to exert anti-inflammatory effects.

## Supplementary Information

ESM 1 (DOCX 883 kb)

## Data Availability

All data and materials supported our published claims and complied field standards.
