# Supplementary material for: Insulin Rescued MCP-1-Suppressed Cholesterol Efflux to Large HDL2 Particles via ABCA1, ABCG1, SR-BI and PI3K/Akt Activation in Adipocytes
Source: Cardiovasc Drugs Ther. 2021 Mar 19;36(4):665–78. doi: 10.1007/s10557-021-07166-2 (PMC9270268; doi:10.1007/s10557-021-07166-2)
Supplement: Supplementary file 1 — (DOCX 883 kb) [file 10557_2021_7166_MOESM1_ESM.docx]

**Insulin rescued MCP-1-suppressed cholesterol efflux to large HDL2 particles via ABCA1, ABCG1, SR-BI and PI3K/Akt activation in adipocytes**

Runlu Sun^1,*^, Pu Fang^2^, Jieyu Jiang^3^, Canxia Huang^4^, Junjie Wang^1,*^, Hongwei Li^1,*^, Xiaoying Wu^1,*^, Xiangkun Xie^1,*^; Yuan Jiang^1,*^, Qian Chen^1,*^, Jinlan Bao^5^, Jingfeng Wang^1^^,*^ Hong Wang^2^ and Yuling Zhang^1,*^

^1^Department of Cardiology, ^4^Intensive Care Unit, ^5^Comprehensive Department, Sun Yat-sen Memorial Hospital, Sun Yat-sen University, Guangzhou, China

^2^Centers for Metabolic & Cardiovascular Research, Department of Pharmacology，Temple University, Philadelphia PA, USA

^3^Graceland Medical Center, the Sixth Affiliated Hospital, Sun Yat-sen University, Guangzhou, China

^*^Guangdong Province Key Laboratory of Arrhythmia and Electrophysiology, Guangzhou, China

Correspondence: Yuling Zhang, Department of Cardiology, Sun Yat-sen Memorial Hospital, Sun Yat-sen University No. 107, the West of Yanjiang Road, Yuexiu District Guangzhou, China, 510120, Email: zzhangyuling@126.com; Hong Wang, Centers for Metabolic & Cardiovascular Research, Department of Pharmacolg，Temple University, Medical Education & Research Building, Rm. 1060 3500 North Broad Street, Philadelphia PA, USA, Email: hongw@temple.edu; Jinfeng Wang, Department of Cardiology, Sun Yat-sen Memorial Hospital, Sun Yat-sen University No. 107, the West of Yanjiang Road, Yuexiu District Guangzhou, China, 510120, Email: wjingf@mail.sysu.edu.cn.

**Table S1** Demographic and clinical characteristics of male CAD patients and healthy controls

|  | **Healthy controls**  **(n=136)** | **CAD patients (n=98)** | ***t/χ^2^*** | ***P*-value** |
| --- | --- | --- | --- | --- |
| Age(years) | 56.91±10.47 | 58.84±9.50 | -1.441^a^ | 0.706 |
| Premature CAD, n (%) | 2(1.5%) | 7(7.1) | 3.540^b^ | 0.060 |
| Hypertension, n (%) | 25(18.4%) | 30(30.6%) | 4.738^b^ | 0.030* |
| Diabetes, n (%) | 0(0) | 34(34.7%) | 55.205^b^ | <0.001* |
| Drinking, n (%) | 7(5.1%) | 7(7.1%) | 0.403^b^ | 0.525 |
| Smoking, n (%) | 29(21.3%) | 29(29.6%) | 2.089^b^ | 0.148 |
| Cr (µmol/L) | 98.29±17.14 | 98.21±12.67 | 0.035^a^ | 0.972 |
| UA (mmol/L) | 324.95±62.37 | 328.27±64.34 | -0.396^a^ | 0.692 |
| FBG (mmol/L) | 4,91±0.65 | 5.30±0.94 | -5.656^a^ | <0.001* |
| TC (mmol/L) | 4.77±1.00 | 5.30±0.94 | -4.-069^a^ | <0.001* |
| TG (mmol/L) | 1.47±0.41 | 2.07±0.87 | -7.029^a^ | <0.001* |
| LDL-C (mmol/L) | 2.98±1.00 | 3.78±0.87 | -6.421^a^ | <0.001* |

Continuous variables were expressed as the means±SD (for normally distributed variables) or medians with interquartile ranges (for non-normally distributed variables). Categorical variables were expressed as the numbers (percentages). CAD, coronary artery disease; Cr, creatinine; UA, uric acid; FBG, fast blood glucose; TC, total cholesterol; TG, triglyceride; LDL-C, low density lipoprotein cholesterol; MCP-1, monocyte chemoattractant protein-1; HDL-C, high density lipoprotein cholesterol; HDL-2C, high density lipoprotein 2 cholesterol; HDL3-C, high density lipoprotein 3 cholesterol; apoA1, apolipoprotein A1. ^a^ represents the *t* statistic; ^b^ represents the *χ^2^* statistic. * *P* <0.05 as significance.

**Fig. S1**


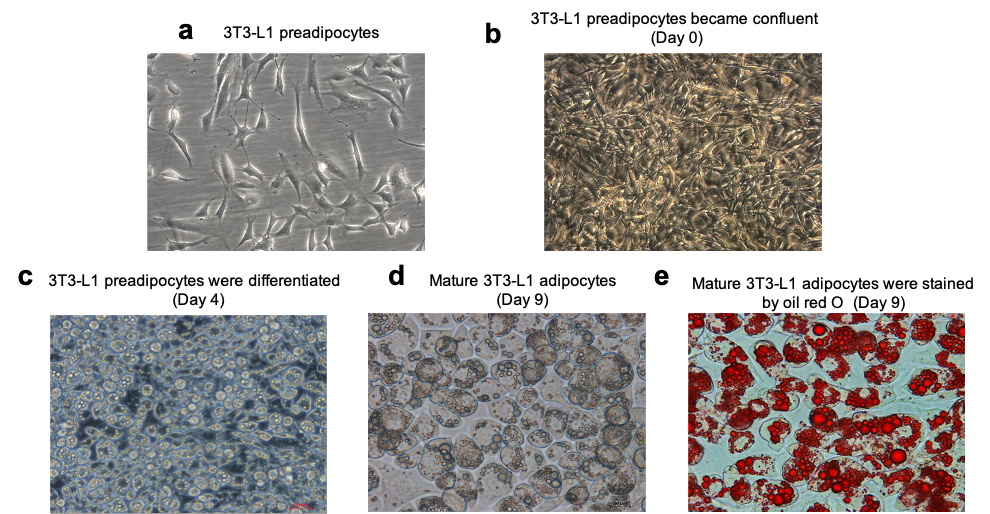


**Fig. S1** 3T3-L1 preadipocytes were differentiated into mature adipocytes by photomicrograph. **(a)** 3T3-L1 preadipocytes (×200). **(b)** 3T3-L1 preadipocytes were confluent after 2 days (Day 0, ×200). (c) 3T3-L1 preadipocytes were differentiated by 1-methyl-3-isobutyl xanthine, dexamethasone and insulin (Day 4, ×200). **(d)** Mature 3T3-L1 adipocytes (Day 9, ×400). **(e)** Mature 3T3-L1 adipocytes were stained by oil red O (Day 9, ×400). Lipid droplets were detected in mature adipocytes.

**Fig. S2**


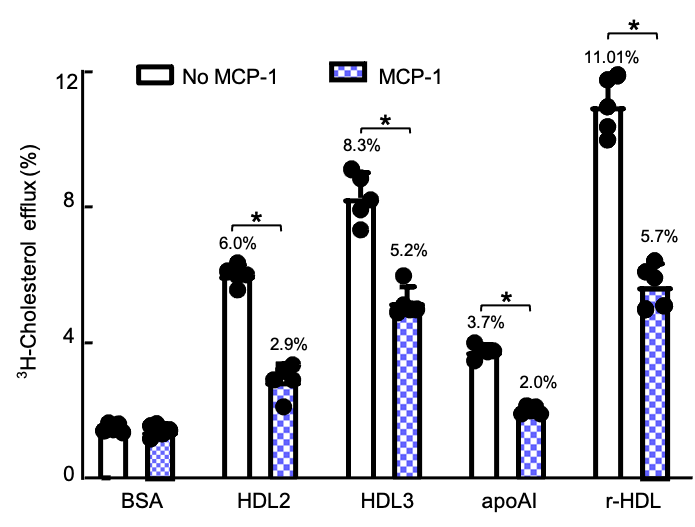


**Fig. S2** MCP-1 suppressed ^3^H-cholesterol efflux to HDL2 and HDL3 particles in differentiated 3T3-L1 adipocytes**.** Fully differentiated adipocytes seeded on collagen-coated 24-well plates were starved for 6 h and labeled with [^3^H]-cholesterol (1Ci/ml) for 24h. Efflux was initiated by 20 µg/ml ApoA-1 or 50 µg/ml HDL2/HDL3/r-HDL (reconstituted HDL) for 2h after cells treated with 40 ng/ml MCP-1 for 48h. ApoA-1 and r-HDL were as the positive control. The radioactivity of the medium and cells was measured with a liquid scintillation counter. The cholesterol efflux was expressed as the percentage of counts in the medium relative to the total counts for the medium and cells together. The results were expressed as mean ± SD (n=5). * *P*<0.05 compared with the untreated cells.
